# Consumers’ Purchase Intention of Organic Food via Social Media: The Perspectives of Task-Technology Fit and Post-acceptance Model

**DOI:** 10.3389/fpsyg.2020.579274

**Published:** 2020-11-05

**Authors:** Jun-Jer You, Din Jong, Uraiporn Wiangin

**Affiliations:** ^1^Department of Exercise and Health Sciences, University of Taipei, Taipei, Taiwan; ^2^Department of Digital Design and Information Management, Chung Hwa University of Medical Technology, Tainan City, Taiwan; ^3^Department of Business Administration, Ramkhamhaeng University, Bangkok, Thailand

**Keywords:** organic food, social media, task-technology fit, post-acceptance model, partial least squares

## Abstract

In the past, consumers were mainly informed about organic food by newspapers, magazines, and television advertisements. However, when consumers buy organic products in the market, they cannot get a complete information about the products from the appearance of the various products. In order to overcome this information asymmetry, social media has become an indispensable part of the promotion of organic food by providing a clear distinction between certified organic products and other types of products in the market. The purpose of this study is to demonstrate the antecedents and consequences of the influence of social media on the consumers’ selection of organic food, based on the post-acceptance model (PAM) and task-technology fit model. The empirical results indicated task characteristics and technology characteristics had the significant effects on confirmation of the expectations and perceived usefulness through the task-technology fit. Besides, the confirmation of expectations and perceived usefulness also influenced significantly the satisfaction and continuance intention, respectively. Finally, the results presented in this article would contribute to the practical and academic implications and recommendations on the promotion of organic food in the social media platform.

## Introduction

For a long time, with the advancement of technology and commercial development, food products have been genetically modified and chemically processed from cultivation to mass production in order to pursue economic benefits while neglecting the potential harm to humans and the environment. As a result, the rise of the consumers’ concept of health and wellness, coupled with the demand for the concept of safe and nontoxic food, has increased the acceptance of organic food, and organic food is gradually becoming a dietary trend (Michaelidou and Hassan, 2008; Benbrook and Baker, 2014; Ronald and Adamchak, 2017). Currently, organic food is a concept and a trend, in addition to the appeal of natural and healthy diet, but it also does not damage the natural environment (farmland, soil, and water), pay attention to the ecology, in order to achieve the principle of symbiotic sustainability between man and nature.

In the past, consumers were mainly informed about organic food by newspapers, magazines, and TV advertisements, and the main motivation for buying organic food is that they feel it is healthier for their health. The value of an organic product lies in the process of production and manufacturing to the point where the final product complies with stringent organic regulations; however, when consumers purchase organic products in the market, they do not get the complete picture from the appearance of the product (Janssen and Hamm, 2012). In addition to its penetration and pervasiveness, social media is also efficient in gathering users of the same type or background (Allagui and Breslow, 2016). According to Palen (2008), survivors of social disasters or victims of major crises are often able to use social media to find others with similar experiences, to share their experiences, and to generate the support and connections that are characteristics of a victim’s community.

Many prior research on social media has been conducted from a technology-acceptance perspective, discussing antecedents and consequences (Chen et al., 2012; Rauniar et al., 2014; Ayeh, 2015; Lin and Kim, 2016; Wamba et al., 2017; Zhao and Wang, 2020). However, the above research results only concentrated on the correlation between the IT and individual usage behavior, and these studies provided a little discussion on how social media influences consumer adoption or purchase intention of organic food. Through this study, we can understand the important factors through which consumers adopt social media to receive messages about organic food. In order to overcome this information asymmetry, social media is playing an important role in the promotion of organic food by clearly separating certified organic products from other types of products in the market and enabling the consumers to identify the labels of certified organic products. At the same time, the transparency of organic food information will increase the consumption of organic food. Therefore, this study devoted effort for developing an integrated model designed to explain and predict the consumers’ purchase intention of organic food based on the concepts of the post-acceptance model (PAM) by Bhattacherjee (2001) and the task-technology fit (TTF) model by Goodhue and Thompson (1995). In addition, previous empirical studies on the impact of organic food purchasing intentions in the past were based on the pre-consumer attitude toward organic food (e.g., Yin et al., 2010; Paul and Rana, 2012; Basha et al., 2015; Singh and Verma, 2017). Therefore, the purpose of this study is to demonstrate the antecedents (three antecedents including task characteristics, technology, and task-technology fit, and two factors including the confirmation of expectations and perceived usefulness) and consequences (two factors including the satisfaction and continuance intention toward organic food social media platform) of the influence of social media on the consumers’ selection of organic food based on the PAM and TTF model using a partial least square (PLS) approach.

## Theoretical Background

### Post-acceptance Model

The expectation confirmation theory (ECT), proposed by Oliver and Richard (1980) and originated in the field of marketing, is based on the concept that (1) consumers had a certain level of expectation for a particular product (or service) before they buy it; (2) after the consumer had experienced the product (or service) for some time, he/she develops a new awareness of the performance that the product (or service) brings; (3) Then, the consumers will compare the perceived performance after experiencing the product (or service) with their initial expectations in order to assess the consistency (i.e., the degree of confirmation); (4) The results of the comparison will affect the level of satisfaction, and the level of satisfaction will affect the likelihood of repurchase or reuse. ECT was extensively used to evaluate consumer satisfaction and behavior after purchase (e.g., repeated purchase and complaint) as well as general service marketing in a past literature review of consumer behavior research (Oliver and Richard, 1980; Tse and Wilton, 1988; Anderson and Sullivan, 1993; Oliver, 1993; Patterson et al., 1997; Dabholkar et al., 2000).

Bhattacherjee (2001) argued that (1) an information system user’s ongoing adoption decision is similar to a consumer’s repurchase decision behavior; (2) affected by the first experience (information system or product); and (3) may eventually reverse the initial decision. Bhattacherjee (2001) argued that the past ECT was somewhat controversial and irrational, and took into account the need to effectively predict and explain the continuous adoption behavior of information system users. Bhattacherjee (2001) modified ECT one by one to make it conform to the use of information systems and proposed the PAM of IS continuance, the main points of which are as follows: (1) PAM focuses on the adoption of the post-acceptance variable because the impact of the adoption of the pre-acceptance variable is covered by the concepts of confirmation and satisfaction. (2) The original ECT only investigated the pre-consumption expectation, but the users’ expectations changed over time, so the postconsumption expectation was especially emphasized in the continued adoption model after the IS acceptance. (3) In the IS acceptance PAM, the post-experience expectations are interpreted as perceived usefulness, a concept consistent with the expectations defined by ECT (i.e., the set of personal beliefs or interbeliefs), and perceived usefulness appropriately represents a user’s significant cognitive beliefs about the information system (Davis, 1989). In recent years, although scholars have been using the PAM to explain the continued adoption of various innovative technologies (e.g., Roca et al., 2006; Bhattacherjee et al., 2008; Chen et al., 2013, 2018; Oghuma et al., 2016; Park, 2020), little is known about the determinants affecting the consumers’ usage intention and evaluation of social media platform to gather relevant information of organic food. Social media operators, which provided and promoted the organic food information should think how to gain rapid acceptance and usage of social media by potential users or consumers. Therefore, this study applied PAM as the basis and extended it to assist social media operators to predict and explain the acceptance of social media platform toward organic foods.

### Task-Technology Fit

Goodhue and Thompson (1995) distinguished the job characteristics in terms of non-routineness and interdependence. Among them, a high level of routine indicates that this type of problem is more likely to be a simple or problematic task. When such tasks are not routine, they tend to be decision-making tasks, judgmental tasks, or vague tasks where the degree of interdependence refers to whether the task can be completed alone or requires the assistance of other departments or other personnel. Tasks with a low level of interdependence may be simple or problematic. Tasks with a high level of interdependence may be problematic, decision making, judgmental, or ambiguous.

The task-technology fit theory proposed by Goodhue and Thompson (1995) emphasizes the impact of technology on individual performance, which is the result of the fit between task, technology, and individual, and an ideal fit can effectively enhance performance. Dishaw and Strong (1999) first attempted to extend task-technology fit and showed that this integration model improved the ability of the model to interpret information technology use. Relevant studies regarding task-technology fit is implemented after Dishaw and Strong (1999) presented the extension of task-technology fit. Klopping and McKinney (2004) studied the propensity and actual purchase behavior of online shopping using an integrated TTF extended TAM model, and the results showed that the degree of interpretable variation of the integrated model was higher than that of the simple TAM model. Larsen et al. (2009) explored information systems through the integration model of the TTF and ECM—the user’s related behavior. Chang (2010) applied the integrated models of a task-technology fit into TAM to evaluate the users’ acceptance of online auctions. Khan et al. (2018) developed an integrated model including the task-technology fit, social motivation, and self-determination theory to represent the technology-oriented, social, and psychological needs of learners regarding the adoption of massive open online courses (MOOCs) in Pakistan.

Therefore, according to the above discussion, the task-technology fit could improve the shortcomings of the PAM and allow this study to take into account the other more relevant factors that influence the consumers’ willingness to purchase organic food through social media.

## Hypotheses Development and Research Methodology

### Research Hypotheses Development

Task-technology fit, defined as how the capabilities of the infrastructure such as information systems match the tasks that the users must perform, is a key determinant in explaining performance levels (Goodhue et al., 2000). The model of task technology, developed by Goodhue and Thompson (1995), has been adopted in various information system/information technology acceptance research (Schrier et al., 2010; Yen et al., 2010; Lu and Yang, 2014; Khan et al., 2018), and it focuses on the matching of the technology to the task, thereby increasing the individual performance (Goodhue et al., 2000). Therefore, we hypothesize the following:

H1: Task characteristics positively affect the perceived task-technology fit in an organic food social media forum.

H2: Technology characteristics positively affect the perceived task-technology fit in an organic food social media forum.

Previous research believed that the perception of whether a particular task-technology fits well with the perceived usefulness could be the basis for constructing perceptions of actually adopting the information technologies (Kim et al., 2010; Wu and Chen, 2017). Empirical evidence has illustrated that the perceived usefulness is affected by the task-technology fit; that is, when the fit between the task and technology is higher, consumers perceive the digital tool to be useful for that task (Larsen et al., 2009; Chang, 2010; Schrier et al., 2010; Lin and Wang, 2012; Wu and Chen, 2017). Moreover, the empirical result from Cheng (2020) also found the positive linage from the task-technology fit to confirmation. Thus, we proposed the following two research hypotheses:

H3: Perceived task-technology fit positively affects the perceived usefulness in an organic food social media forum.

H4: Perceived task-technology fit positively affects the confirmation in an organic food social media forum.

Satisfaction is critical to promote the successful implementation toward information systems/information technologies (Au et al., 2008). Therefore, satisfaction plays a critical role in PAM (Bhattacherjee, 2001; Chen et al., 2013, 2018). Prior research evidently supported the confirmation of expectations and perceived usefulness continuance intention through satisfaction toward various digital and mobile services (Lin et al., 2005; Chen et al., 2013; Oghuma et al., 2016; Park, 2020; Tam et al., 2020). According to the above evidence, a summary of research hypotheses related to PAM and intention to use social media in gathering organic food information environment are presented as follows:.

H5: Confirmation positively affects the perceived usefulness in an organic food social media forum.

H6: Perceived usefulness positively affects the continuance intention in an organic food social media forum.

H7: Perceived usefulness positively affects the satisfaction in an organic food social media forum.

H8: Confirmation positively affects the satisfaction in an organic food social media forum.

H9: Satisfaction positively affects the continuance intention in an organic food social media forum.

### Research Methodology

Considering the above nine hypotheses, we designed a questionnaire to measure and understand the users’ perceptions in the organic food social media forum. For content validity, all the measurement items for each latent variable in the questionnaire were from existing studies. PAM, including the continuance intention, satisfaction, perceived usefulness, and confirmation, were adopted from Bhattacherjee (2001) and Chen et al. (2013). The concept of the task-technology fit, including the technology characteristics and task characteristics, were slightly modified from Chang (2010) and Wu and Chen (2017). Each measurement item was replied following the seven-point Likert’s scale. For example, range from 1, “strongly disagreement” to 7, “strongly agreement.”

**FIGURE 1:**
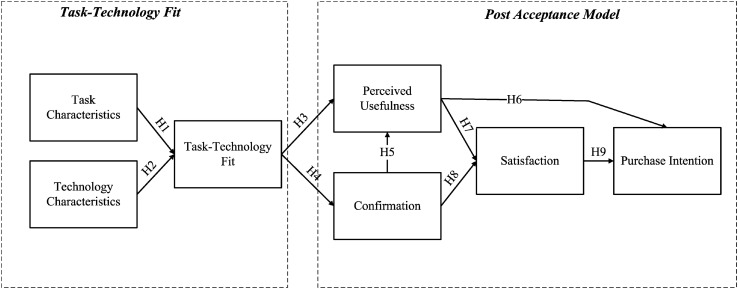
Integration model of task-technology fit (TTF) and post-acceptance model (PAM).

In this study, the partial least squares (PLSs) is used as the data analysis tool for the research model. The PLS is a structural equation modeling (SEM) analysis technique based on a regression analysis, which is a statistical method derived from the path analysis. By using PLS analysis, both the measurement model of the research tool and the structural model of the research component can be examined. Past research has shown that PLS can evaluate both the measured model and the theoretical structural model and is, therefore, superior to the traditional regression analysis and factor analysis methods (Urbach and Ahlemann, 2010; Hair et al., 2012; Henseler et al., 2015). In addition, compared with the LISREL method commonly used in the academia, PLS requires a smaller sample size for analysis, and the observational data is not available. PLS requires multivariate constant assumptions and is better at prediction. Model flexibility is also greater (Ringle et al., 2012). PLS analysis software used in this study is the SmartPLS (version 3.3.2) and uses bootstrap resampling method to check the significance of the paths in the structural model. The research hypotheses of this research are shown in Table 1, and the research framework is shown in Figure 1.

**TABLE 1:** Research hypotheses.

Hypothesis	Hypothesis statement
Hypothesis 1	Task characteristics positively affect the perceived task-technology fit in an organic food social media forum.
Hypothesis 2	Technology characteristics positively affect the perceived task-technology fit in an organic food social media forum.
Hypothesis 3	Perceived task-technology fit positively affects the perceived usefulness in an organic food social media forum.
Hypothesis 4	Perceived task-technology fit positively affects the confirmation in an organic food social media forum.
Hypothesis 5	Confirmation positively affects the perceived usefulness in an organic food social media forum.
Hypothesis 6	Perceived usefulness positively affects the continuance intention in an organic food social media forum.
Hypothesis 7	Perceived usefulness positively affects the satisfaction in an organic food social media forum.
Hypothesis 8	Confirmation positively affects the satisfaction in an organic food social media forum.
Hypothesis 9	Satisfaction positively affects the continuance intention in an organic food social media forum.

A questionnaire survey was used to collect data by convenience sampling method, and questionnaires were collected from four organic food promotion Facebook communities. To ensure the content validity, all samples had relevant experience in participating in organic food discussions or purchasing behavior through social media. The sample recall period was 4 weeks. The total number of samples collected was 284. After data checking and deletion of invalid questionnaires, the remaining number of valid samples was 235. Among them, 41.2% were male and 58.8% were female. Sixty-two percent (60.2%) of the samples had university education, and most of them (72.6%) were aged 26 to 40 years old.

## Data Analysis

### Outer Model

In order to further examine the reliability and validity of the scale questionnaire, this study uses the PLS statistical software to check the appropriateness of the measurement model. The validation of the measurement model includes checks for internal consistency, convergent validity, and discriminate validity. Reliability is the correctness and accuracy of a measurement tool, which has two meanings. Stability is the degree of reliability of retesting and consistency is the measurement of the internal consistency among the items in the questionnaire. This study adopts the composite confidence level of the least square method (PLS). Reliability was analyzed with the Cronbach’s α and composite reliability. According to Fornell and Larcker (1981), the composite reliability (CR) and Cronbach’s α should be above 0.7 to ensure that the measurement variables are internally consistent. While the CR values in this study were all above 0.880 (as shown in Table 1), the Cronbach’s α values were all above 0.728 and above, indicating that the internal consistency of the measurement tools designed in this study is acceptable.

Convergent validity indicates the degree to which multiple variables measure the same dimension. According to the suggestion of Fornell and Larcker (1981), the average variance extracted (AVE) for each individual component must be greater than 0.5, and the factor loadings of each component must be greater than 0.5 (Hair et al., 2010). The factor loadings of each component must be greater than 0.5 to be considered as having sufficient convergent validity. The AVE values of each structure are above 0.699 (as shown in Table 2). According to the above analysis, it means that the measurement model in this study passes the test and satisfies the need for the convergent validity (as shown in Table 2).

**TABLE 2:** Reliability and convergent validity.

Construct	Cronbach’s alpha	Composite reliability	AVE
CI	0.890	0.932	0.821
CON	0.919	0.943	0.804
PU	0.901	0.931	0.771
SAT	0.935	0.959	0.885
TASK	0.846	0.907	0.764
TECH	0.857	0.903	0.699
TTF	0.728	0.880	0.786

*AVE, average variance extracted. CI, continuance intention; CON, confirmation; PU, perceived usefulness; SAT, satisfaction; TASK, task characteristics; TECH, technology characteristics; TTF, Task Technology fit.*

The discriminant validity is to check how well the measurement variables discriminate between the different configurations. The correlation between each variable and other variables of the same dimension should be higher than the correlation between the variables of the different dimensions. In order to pass the test of discriminant validity, the square root of AVE from an individual component should be greater than the correlation coefficient (non-diagonal value) between that component and the other components in the model to indicate the discriminant validity (Fornell and Larcker, 1981). Table 3 shows the matrix of correlation coefficients between each component, and the diagonal lines are the square roots of the AVE of that component. Table 3 shows that the square roots of the AVEs for each of the structural measurement items are larger than the correlation coefficients between the two components, which means that the questions of different components in the questionnaire of this study can be discriminated adequately. In addition, by comparing the factor loadings and cross loadings within each study construct, it can also be seen that this study has a good discriminant validity (as shown in Table 4).

**TABLE 3:** Correlations and discriminant validity.

Construct	CI	CON	PU	SAT	TASK	TECH	TTF
CI	**0.906**						
CON	0.734	**0.897**					
PU	0.558	0.680	**0.878**				
SAT	0.714	0.835	0.598	**0.941**			
TASK	0.546	0.601	0.625	0.640	**0.874**		
TECH	0.527	0.575	0.474	0.610	0.666	**0.836**	
TTF	0.523	0.582	0.618	0.557	0.659	0.537	**0.887**

*CI, continuance intention; CON, confirmation; PU, perceived usefulness; SAT, satisfaction; TASK, task characteristics; TECH, technology characteristics; TTF, task technology fit. The diagonal value represents the square root of the AVE. The nondiagonal value represents the correlation value of each component. The bold values means the square roots of the AVE.*

**TABLE 4:** Factor loadings and cross loadings.

	CI	CON	PU	SAT	TASK	TECH	TTF
CI1	**0.927**	0.643	0.541	0.635	0.480	0.463	0.445
CI2	**0.939**	0.656	0.545	0.616	0.495	0.425	0.477
CI3	**0.849**	0.693	0.430	0.686	0.507	0.540	0.496
CON1	0.627	**0.892**	0.606	0.695	0.511	0.524	0.478
CON2	0.643	**0.905**	0.598	0.710	0.514	0.512	0.531
CON3	0.620	**0.892**	0.628	0.807	0.544	0.491	0.489
CON4	0.738	**0.898**	0.606	0.777	0.583	0.536	0.585
PU1	0.482	0.529	**0.839**	0.445	0.515	0.385	0.544
PU2	0.429	0.551	**0.885**	0.482	0.560	0.383	0.597
PU3	0.475	0.622	**0.921**	0.543	0.565	0.445	0.572
PU4	0.563	0.671	**0.865**	0.612	0.553	0.446	0.468
SAT1	0.639	0.734	0.525	**0.921**	0.571	0.519	0.482
SAT2	0.680	0.794	0.579	**0.963**	0.615	0.593	0.545
SAT3	0.695	0.826	0.581	**0.938**	0.619	0.605	0.542
TASK1	0.509	0.520	0.584	0.569	**0.879**	0.570	0.614
TASK2	0.480	0.573	0.563	0.592	**0.887**	0.586	0.526
TASK3	0.440	0.488	0.490	0.520	**0.856**	0.592	0.581
TECH1	0.479	0.473	0.424	0.486	0.560	**0.806**	0.526
TECH2	0.320	0.348	0.316	0.404	0.473	**0.827**	0.336
TECH3	0.457	0.514	0.378	0.523	0.538	**0.894**	0.417
TECH4	0.463	0.550	0.435	0.595	0.624	**0.814**	0.467
TTF1	0.458	0.525	0.541	0.497	0.630	0.488	**0.893**
TTF2	0.469	0.507	0.555	0.491	0.537	0.464	**0.880**

*CI, continuance intention; CON, confirmation; PU, perceived usefulness; SAT, satisfaction; TASK, task characteristics; TECH, technology characteristics; TTF, task technology fit. The bold values means standardized factor loadings.*

### Inner Model

**FIGURE 2:**
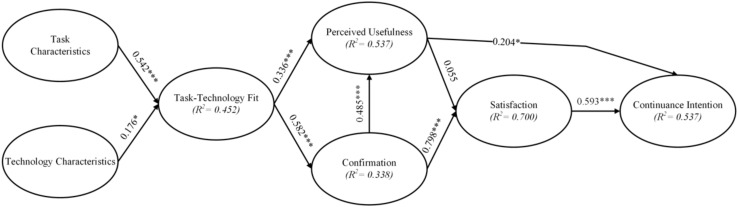
Inner model.

In this study, PLS is used as an analytical tool to analyze the strength and direction of the relationship between the study variables in the structural model. The structural model is validated mainly in terms of the estimated path coefficients and *R*-values. The coefficient of path represents the strength and direction of the relationship between the study variables and should be verified as significant and consistent with the expected direction of the hypothesis. The *R*-value is the percentage of variation explained by the exogenous variables versus the endogenous variables and represents the predictive power of the research model. The path coefficients and *R*-values together show how well the structural model and data fit together. The results of the analysis of the structural model of this study are shown in Figure 2 and Table 5.

**TABLE 5:** Inner model and path analysis.

	Original sample (O)	Standard deviation (STDEV)	*T* Statistics (|O/STDEV|)	*P*-values
H1: TASK → TTF	0.542	0.080	6.747	0.000
H2: TECH → TTF	0.176	0.082	2.154	0.031
H3: TTF → PU	0.336	0.079	4.262	0.000
H4: TTF → CON	0.582	0.060	9.690	0.000
H5: CON → PU	0.485	0.083	5.858	0.000
H6: PU → CI	0.204	0.083	2.462	0.014
H7: PU → SAT	0.055	0.068	0.807	0.420
H8: CON → SAT	0.798	0.058	13.839	0.000
H9: SAT → CI	0.593	0.067	8.828	0.000

*CI, continuance intention; CON, confirmation; PU, perceived usefulness; SAT, satisfaction; TASK, task characteristics; TECH, technology characteristics; TTF, task technology fit.*

Each component affects the part of the persistent use of intention, including the perceived usefulness (β = 0.204, *p* < 0.014) and satisfaction (β = 0.593, *p* < 0.001); both of these two constructs have a significant effect on the persistence of the intention. The effect of partial confirmation of the satisfaction (β = 0.798, *p* < 0.001) on the satisfaction was significant, but the perceived usefulness was not. The effect of β (β = 0.055, *p* = 0.420) on the satisfaction was not significant. In the section on the perceived usefulness of each component, the confirmation (β = 0.485, *p* < 0.001) and task-technology fitness (β = 0.336, *p* < 0.001), both components had a significant effect on the perceived usefulness. Task-technology fit (β = 0.582, *p* < 0.001) has a significant effect on the degree of recognition. Finally, in the component of task-technology fitness for each construct, the task characteristics (β = 0.542, *p* < 0.001) and technology characteristics (β = 0.176, *p* < 0.05), the effect of the task-technology fit is significant for both structures.

## Discussion and Conclusion

The study contributes to the theoretical assessment by integrating PAM and TTF as the research framework to investigate the factors that influence the consumers’ adoption of organic food information through social media. Our study adopted the PAM presented by Bhattacherjee (2001) as the framework basis, adds TTF as the external variable, and establishes a research framework to understand the influence of PAM on the consumers’ purchase intention toward organic food via social media. This framework analyzes the factors when the users use cloud computing services, such as TTF, perceived usefulness, confirmation, satisfaction, and continuance intention, to assess the influence of TTF on the continuance intention. After that, the data were collected through questionnaires, and the model was validated using the PLS method, and many significant findings were obtained. Compared with the individual TTF and PAM models, the integrated model provides more explanation power on the consumers’ adoption toward an organic food social media platform. The proposed model of this study provides more precise results than the PAM and TTF model do separately, and the extended model assists practitioners and scholars in understanding the dynamics of organic food information toward social media platform.

The purpose of this survey was to understand and validate the proposed model of integrating PAM and TTF to assess the influencers of the consumers’ continuance intention to gather the information regarding organic food in social media environment. Important findings were outlined as follows. First, as theorized by TTF, both task characteristics and technology characteristics significantly influence the task-technology fit. TTF, by itself, is also a robust model in which the task and technology characteristics significantly determine the correspondence between information acquisition of organic food (i.e., task requirements) and technology functionality of social media platform. In addition, the task characteristics were found to be more influential than the technology characteristics. By decomposing TTF, we offer relevant perspectives as to which task and technology dimension becomes critical in the consumers’ acceptance decisions for the organic food social media platform. Second, for the PAM model, except for the perceived usefulness to satisfaction, the other four paths—the perceived usefulness and satisfaction to continuance intention, the confirmation to perceived usefulness and satisfaction—have positive and significant impacts. Because satisfaction is the most critical determinants of the continuance intention (explaining 70% of variance) relative to the other determinants, the consumers dissatisfied with an organic food social media platform may stop using it, despite having positive perceptions with regard to other factors. Third, we also found that there exists correlations between the TTF and PAM ones (i.e., confirmation of expectations and perceived usefulness). In recent years, due to changes in dietary patterns, the number of people eating out has increased, resulting in many health problems caused by excessive and unbalanced diets. In addition, the pesticides and chemicals used by farmers in the past to grow crops with beautiful appearance and integrity have seriously contaminated the soil, water, and air, leaving a large amount of harmful substances on the surface of the crops or inside the organisms, which can cause diseases. Therefore, understanding how to find and promote organic food information and food safety through social media is an important issue in the relevant research area. All countries have strict organic product labeling regulations and state that only products that comply with national organic regulations can be sold with organic labels or organic names, and prohibit other products that do not comply with organic regulations from using labels that mislead consumers into thinking that the product is organic.

## Research Limitations and Future Work

This study strives to be rigorous, objective, and thorough in its process and analysis in order to obtain good research results. However, due to time, manpower, and environment-related factors, there are still the following research limitations. First, the sample of this study was drawn from consumers who purchased organic food through social media, which was sufficient to ensure the content validity. However, in the future, it is recommended that a consumer segmentation analysis should be conducted on different ethnic groups, consumption patterns, and habits in order to better understand the pre-purchase factors of different consumer groups for organic food. Second, cost-related factors have not been included in this study. There is little literature on the cost of promoting organic food behaviors through social media, and most of the literature on the price and behaviors is in the marketing research area. Therefore, we suggest that the follow-up study can be extended to examine how the cost and willingness to pay for organic food through social media affects the consumers’ purchasing behavior.

## Data Availability Statement

The raw data supporting the conclusions of this article will be made available by the authors, without undue reservation.

## Ethics Statement

Ethical review and approval was not required for the study on human participants in accordance with the local legislation and institutional requirements. Written informed consent from the patients/participants or patients/participants legal guardian/next of kin was not required to participate in this study in accordance with the national legislation and the institutional requirements.

## Author Contributions

J-JY and DJ developed the original idea for the study, contributed to research design, and performed the sample collection and data analysis. J-JY, DJ, and UW wrote the manuscript. All authors read and approved the final manuscript.

## Conflict of Interest

The authors declare that the research was conducted in the absence of any commercial or financial relationships that could be construed as a potential conflict of interest.
